# A qualitative analysis of psychosocial outcomes among women with sexual violence-related pregnancies in eastern Democratic Republic of Congo

**DOI:** 10.1186/s13033-017-0171-1

**Published:** 2017-10-18

**Authors:** Jennifer Scott, Colleen Mullen, Shada Rouhani, Philipp Kuwert, Ashley Greiner, Katherine Albutt, Gillian Burkhardt, Monica Onyango, Michael VanRooyen, Susan Bartels

**Affiliations:** 1 Department of Obstetrics and Gynecology, Beth Israel Deaconess Medical Center, 330 Brookline Avenue, Kirstein 3rd Floor, Boston, MA 02215 USA; 2 Division of Women’s Health, Brigham and Women’s Hospital, 75 Francis Street, Boston, MA 02115 USA; 3 Harvard Humanitarian Initiative, 14 Story Street, Cambridge, MA 02138 USA; 4 Harvard Medical School, 25 Shattuck Street, Boston, MA 02115 USA; 5 Department of Psychiatry, One Boston Medical Center Place, Boston Medical Center, Boston, MA 02118 USA; 6 Department of Psychiatric Emergency Services for Cambridge/Somerville, Boston Medical Center, Boston, MA USA; 7 Department of Emergency Medicine, Brigham and Women’s Hospital, 75 Francis Street, Boston, MA 02115 USA; 8 Department of Emergency Medicine, Beth Israel Deaconess Medical Center, 190 Pilgrim Road, Boston, MA 02215 USA; 9 Department of Surgery, Massachusetts General Hospital, 55 Fruit Street, Boston, MA 02114 USA; 10 Department of Psychiatry and Psychotherapy, and HELIOS-Hansehospital Stralsund, University of Greifswald, Greifswald, Germany; 11 Harvard School of Public Health, 677 Huntington Avenue, Boston, MA 02115 USA; 12 Department of Emergency Medicine, Queen’s University, 76 Stuart Street, Kingston, ON K7L 2V7 Canada; 13 Department of Global Health, Boston University School of Public Health, 801 Massachusetts Avenue, 3rd floor, Boston, MA 02118 USA; 14 Department of Obstetrics and Gynecology, Boston University School of Medicine, 85 East Concord Street, Boston, MA 02115 USA; 15 Department of Obstetrics and Gynecology, University of New Mexico, MSC 10 5582, Albuquerque, NM 87131 USA

**Keywords:** Sexual violence, Pregnancy, Mental health, Stigma, Psychosocial outcomes, Social support

## Abstract

**Background:**

Sexual violence is prevalent in eastern Democratic Republic of Congo (DRC) and has potentially devastating psychosocial consequences. Previous studies have reported on sexual violence and its impact on the mental health of survivors, but there are few studies conducted among women with sexual violence-related pregnancies (SVRPs). Women with SVRPs may be at greater risk of complex psychosocial outcomes, including social stigmatization. This study aimed to describe psychosocial outcomes among this subgroup of sexual violence survivors in order to inform future interventions.

**Methods:**

A mixed methods study was conducted in Bukavu, DRC in 2012 among adult women who self-reported an SVRP and either (1) were currently raising a child from an SVRP (parenting group) or (2) had terminated an SVRP (termination group). This manuscript presents qualitative findings from the mixed methods study. Participants were recruited using respondent-driven sampling and a proportion engaged in semi-structured qualitative interviews conducted by trained female interviewers. Thematic content analysis was conducted and key themes were identified.

**Results:**

In total, 55 women were interviewed, of whom 38 were in the parenting group and 17 in the termination group. Women with SVRPs experienced a myriad of emotional responses as they navigated their social environments following the SVRPs. Negative reactions, including social stigmatization and/or social rejection, toward women with SVRPs and toward children born from SVRPs were important influences on psychological well-being. Women expressed both internalized emotionality intertwined with externalized experiences in the social environment. Many women demonstrated resilience, or what could be termed post-traumatic growth, identifying avenues of agency to advance the social conditions for women.

**Conclusions:**

The findings from the qualitative study, and in particular, the respondents’ needs and suggested strategies, may be useful to inform future research, programs, and policies for women with SVRPs in eastern DRC. Future research could move beyond cross-sectional assessments to utilize innovative research methodologies to assess processes of psychological adaptation among women with SVRPs. Multi-dimensional psychosocial programs for women with SVRPs should consider basic needs such as shelter, food, and health care within the broader framework of trauma-informed care. Participatory programming, guided by beneficiaries, could provide further avenues for agency to advance social conditions for women with SVRPs in eastern DRC.

## Background

Sexual violence, whether perpetrated by militarized actors or non-combatants, is prevalent in eastern Democratic Republic of Congo (DRC) and impacts the psychosocial well-being and mental health of survivors [1–4]. In eastern DRC, up to one in five sexual violence survivors has reported a sexual violence-related pregnancy (SVRP) [3, 5, 6]. Women with SVRPs may be at greater risk of complex psychosocial sequelae [5, 7–10]; however, few studies focus on this subgroup of sexual violence survivors.

In previous studies on sexual violence in eastern DRC, survivors have reported symptoms of depression, post-traumatic stress disorder (PTSD), anxiety, and suicidality [3, 11, 12]. Survivors have also described social stigmatization, exclusion, and rejection as a result of sexual violence [7, 8, 13]. Social stigmatization has been noted to be a mediating factor for mental health outcomes among survivors [14, 15], highlighting the influence of social factors on mental health. Previous research has demonstrated that social support is important in post-trauma adjustment after sexual violence [16, 17]; as such, negative social reactions may impede post-traumatic growth [18]. While negative social reactions toward sexual violence survivors, such as stigmatization, have been shown to influence mental health and severity of trauma-related symptoms [19–21], there are limited data on psychosocial outcomes among women with SVRPs.

Previous quantitative studies of mental health and psychosocial consequences provide a platform from which to appreciate the complexities of assessing mental health on a global level [22, 23]. However, there are noted limitations of these assessments including limited cultural validity and constraints on discerning and inclusion of other psychosocial factors that may influence mental health. Furthermore, mental health assessments conducted post-trauma are often focused on the traumatic event, such as sexual violence, and fewer assess how the social environment influences psychological well-being following the trauma [19, 20]. Qualitative data can provide further understanding of individuals’ experiences and inform mental health and psychosocial support interventions for sexual violence survivors in conflict settings and women with SVRPs [12, 24–26].

Finally, further understanding of the experiences of women with SVRPs may also inform broader reproductive health programs and policies. Reproductive options for women with SVRPs are limited in DRC, where pregnancy termination, even in the context of sexual violence, is criminalized [27]. There is a growing focus on children born from SVRPs [28] and understanding the experiences of their mothers may add further dimension to the dialogue on this issue. As part of a larger mixed methods study of women with SVRPs in eastern DRC [29], a qualitative study was conducted to describe their emotional responses, needs, and psychosocial outcomes.

## Methods

### Study design

A mixed methods study was conducted in October–November 2012 in Bukavu, DRC. The study applied quantitative and qualitative methods to assess outcomes among adult women (18 years and older) who self-identified as survivors of sexual violence since the start of the war (~ 1996) and conceived pregnancies as a result of sexual violence. There were two study groups: (1) women currently raising children from SVRPs (parenting group) and (2) women who had terminated SVRPs (termination group). Recruitment and participation in study groups was not mutually exclusive, such that women in the parenting group could recruit women who had terminated a pregnancy and vice versa, and also to recognize that some participants may have had more than one lifetime SVRP and had different outcomes from those SVRPs. Previous publications from this study present quantitative data on mental health and parenting outcomes [30, 31], as well as quantitative and qualitative data on pregnancy termination [31, 32].

### Sampling

Women were recruited to participate in the study using respondent-driven sampling (RDS), a method designed to sample hard-to-reach populations [33]. Respondent-driven sampling uses a peer recruitment sampling system that allows for the estimation of sample weights to correct for biases associated with traditional chain referral sampling [33]. Further details on the use of RDS in this study were previously published [29].

### Study procedures

Initial study participants who met study eligibility criteria were identified through partner organizations in Bukavu and interviewed. Upon completing interviews, each initial participant received uniquely numbered coupons to recruit up to three peers who met study criteria. Peers presented to the study office where study eligibility was assessed, interviews were conducted, and each participant in turn received uniquely numbered coupons to recruit up to three other peers.

A proportion of participants were invited to participate in a semi-structured interview after completing the quantitative survey. We anticipated lower recruitment and enrollment in the termination group of the larger mixed methods study compared to the parenting group because of the sensitivities related to pregnancy termination in DRC; therefore, every 5th participant in the termination group and every 20th participant in the parenting were invited to complete an interview. The interviews were conducted after the quantitative survey and no one dropped out or refused to participate in a qualitative interview. All participants received a referral card for medical and/or psychosocial care. Transportation was reimbursed and participants received a headscarf as a token of appreciation (~ $1 USD).

### Interviewer selection and training

Interviewers were recruited from the local area and were selected based on previous experience working with sexual violence survivors and/or conducting research on sensitive subjects such as sexual violence. Three researchers (SB, JS, KA) conducted an intensive, on-site 6-day interviewer training on research aims, methodology, recruitment, interviewing techniques, and ethics. In addition, daily debriefings were held with the interviewers during data collection. Recognizing that interviewers may have certain attitudes or beliefs about women with children from SVRPs or about women who terminated an SVRP, potential sources of biases and the importance of neutrality were emphasized during the training and debriefings. Interviewers did not have a relationship with study participants prior to study commencement.

### Interview procedures

Verbal consent was obtained from participants due to varied literacy levels among the study population and to minimize potential risks to participants from written documentation of their participation in the study. Interviews were conducted in private rooms at the study office in Bukavu, with only the interviewer and participant present. In keeping with gender-based violence research standards [34], children over the age of two were not present. For the qualitative study, interviewers verbally administered qualitative survey instruments specific to each study group; qualitative interviews lasted approximately 30–45 min. The questions focused on experiences related to the SVRP and not on the sexual violence incident itself, although open responses were encouraged. Participants were also asked to comment on their concerns in general and what would be helpful for women in DRC, women with SVRPs, and children born from SVRPs. The instruments were written in English, translated into Kiswahili, and back translated to English by a different translator. Translation differences were resolved by consensus with a third translator and local collaborators verified accuracy and clarity of the translated questions.

The interviewers recorded responses by hand in Kiswahili during the interview, but no other notes were collected to minimize risk to participants. Following the interview, a trained interpreter in DRC translated the handwritten responses into English and created electronic files. To preserve participant privacy, no identifying information was collected, and participants were not contacted or re-interviewed following the completion of the initial interview. The study terminated in November 2012 due to security reasons.

### Ethical considerations

The institutional review board at the Harvard School of Public Health provided human subjects research approval. The medical inspector in South Kivu Province granted permission to conduct the study. A community advisory board in Bukavu provided study oversight. No identifying information was collected and the study name and documentation did not disclose the nature of the study. The interviewer training included dedicated sessions on a trauma-informed approach to interviewing and how to recognize symptoms of psychological distress. A psychosocial assistant was present in the study office at all times, and there was a referral procedure for the study if additional medical and/or psychosocial resources were needed. The manually recorded interviews were stored securely in the study site and the electronic files were password protected.

### Data analysis

The electronic files were uploaded to the qualitative data analysis software, Dedoose (Version 5.0.11, Los Angeles, CA). Thematic content analysis was applied to identify themes and patterns in the text data [35, 36]. Two researchers (CM, SB) conducted an initial review of the transcripts to determine preliminary codes for thematic organization of the data. These preliminary codes were then further refined by two researchers (JS, SB). Data were independently coded by JS and SB and organized into key conceptual themes. Coding inter-rater reliability was measured with a pooled Cohen’s kappa (0.92) [37].

The overall coding framework for the qualitative study comprised the following categories (Table 1): psychosocial responses, disclosure about the pregnancy, social and contextual determinants of decision-making related to the SVRP, and access to abortion services. Within the psychosocial responses category, there were the following codes, each with multiple subcodes: emotional responses, strategies for responding to a situation, resilience, pride and respect, request for services and aid, and a call for peace. Throughout the process, researchers triangulated the qualitative data with the previously analyzed quantitative data, the existing literature, and peer debriefing. Preliminary findings were presented to study partner organizations in-person in Bukavu.

**Table 1:** Coding framework for qualitative study and psychosocial analysis

Overall coding framework for study	Sub-codes for psychosocial analysis
Psychosocial responses	Emotional responses
Disclosure of the pregnancy	Strategies for responding to a situation
Decision-making related to the SVRP	Resilience
Access to abortion services	Pride and respect
	Request for services and aid
	Call for peace

*SVRP* sexual violence-related pregnancy

## Results

In total, 55 participants were interviewed for this qualitative study, of whom 38 participants were from the parenting group and 17 participants from the termination group. The mean age of participants at the time of the study was 33.7 years (18–60 years) and marital status at the time of the study was reported as divorced or separated from spouse (29%), widowed (24%), married (20%), or never married (20%). A majority (87%) reported little or no formal education. Participants represented the ethnic (Bashi 79%) and the religious majorities (Catholic 54%, Protestant 46%) in Bukavu. Major themes identified in the qualitative data analysis and excerpts from interviews are presented below.

### Theme 1: Emotional responses and psychological well-being among women with SVRPs

When participants were asked to describe their reactions to finding out about the SVRP, they reported a myriad of emotional responses. The predominant emotional response expressed in both study groups was sadness, which often manifested as cyclic weeping (emotional dysregulation) and disrupted sleep, among other symptoms. One 22-year-old married woman raising a child from an SVRP described experiencing sadness and generalized disinterest during her pregnancy:

When I realized that I didn’t have my period, I was sad. I was rejected and hated because friends wanted me to perform termination. I used to cry every day and hardly got to sleep. I disliked everything. I didn’t take any liking for something from the first month to the ninth. I could not laugh. I didn’t want to meet people, I stayed at home.

In many cases, sadness co-existed with feelings of anxiety, restlessness, and anger. Restlessness was more commonly described in relation to the decision-making process related to the SVRP. A few women reported feeling angry as a result of the SVRP. For example, one woman raising a child from an SVRP reported feeling anger toward herself, while other women in the termination group cited generalized feelings of anger as reasons for deciding to terminate the SVRP. A 22-year-old married woman who terminated an SVRP described her experience:

I was raped for the second time in February 2012… I realized that I was pregnant at the end of May…I felt hopeless, nervous, and angry. I took the money I had and went to see a nurse to help me terminate the pregnancy.

Another predominant reaction to the SVRP was an overwhelming sense of social isolation, both self-induced and enforced by their social environments. Shame, commonly reported as a reason for social isolation from their families and friends, was described among women in both study groups. A 60-year-old widowed woman who was raising a child from an SVRP reported:

I’m a widow…When I realized I was pregnant from sexual violence I was so restless and about to die of a broken heart. I would be ashamed in case my children learned I was pregnant. I was nervous because I was very old and weak and I’ve already stopped giving birth. I didn’t inform anybody about the pregnancy. I hid myself in the house weeping.

When asked about the reaction to finding out about the SVRP, suicidality was reported among seven women in the parenting group; however, it was not reported among women in the termination group. In the parenting group, suicidal ideation and attempts were most commonly mentioned in relation to being rejected by her spouse, family, or community. A 28-year-old widowed woman from the parenting group described her experience as follows:

When I realized I was pregnant from sexual violence, I attempted suicide. I took an overdose of medicine but I failed.

### Theme 2: Social influences on psychological well-being for women with SVRPs

When asked about their experiences with the SVRP, including to whom they disclosed the SVRP and the decision-making related to the SVRP, women mostly described challenges coping within their social contexts. Numerous themes emerged regarding social consequences of SVRPs, including anticipated or perceived social stigmatization and social rejection.

In many cases, women reported how the reactions of spouses, family members, and community members influenced their emotional responses to the SVRP. Women expressed distress associated with being abandoned and rejected by their spouses. There were also references to anticipated loss of future social opportunities and social stability, such as marriage, as women considered their futures as survivors of violence and as women with SVRPs within their social contexts. One 31-year-old woman in the parenting group, who reported being abandoned after experiencing an SVRP, expressed her feelings of isolation in the community due to stigma:

When somebody wants to woo me, people let him know that I’m the wife of [armed group]. I feel restless and stigmatized in my community that I need to leave it…In the future, my main worry is the negative attitude towards women victims of sexual violence in DR Congo. Other women laugh at them.

Social stigmatization and rejection were commonly reported among the parenting group, but they were also cited as reasons for considering pregnancy termination. In some cases it is clearly articulated that the stigmatization and/or rejection are attributed to the SVRP, but in other cases, women did not articulate in the interviews whether the rejection resulted from the incident of sexual violence, the SVRP, or another consequence of sexual violence. A 32-year-old woman in the parenting group who was separated from her husband reported perceived stigma and the potential impact on her future:

I was married, but now I’m separated from my spouse…I was so restless and felt ill. I couldn’t imagine what people were thinking of me…My concern is that I’m separated from my spouse, no chance to get married when I’m stigmatized by people.

Community rejection and stigmatization resulting in migration were also reported, as a 33-year-old widowed respondent in the parenting group detailed:

When I was taken to the bush my spouse was killed. We were with our three children, one passed away and two remained (a boy and a girl). Then I gave birth to twins, two boys from sexual violence. Presently, I am living with my sister because I was rejected by the community in my village.

### Theme 3: Social stigmatization and/or social rejection toward children born from SVRPs

In addition to the anticipated or perceived social stigmatization and/or rejection experienced by women as a result of SVRPs, women also described stigmatization toward their children born from SVRPs. Women expressed concerns about their spouses’ treatment of children born from SVRPs, such as controlling or denying financial or material resources for the child. A 25-year-old woman from the parenting group who was abandoned by her husband reported her concerns over spousal rejection of a child from sexual violence, and the financial insecurity related to stigma:

When we returned home in the village, my spouse rejected me. Two months later, I realized I was pregnant. I felt on edge because my spouse rejected me, but I decided to carry the pregnancy to term…My concern is that my spouse stigmatizes that child from sexual violence. I wonder if he will support school charges. Women undergo hardships… sexual violence is a big issue, children are rejected.

Women also identified the need for further social and financial support for children born from SVRPs in DRC. Given the nature of the study groups, stigmatization toward children was more commonly expressed among the parenting group; however, qualitative data from the termination group also indicate that anticipated stigmatization of children born from SVRPs influenced the decision to terminate the SVRP. One 60-year-old widow in the termination group described her reasoning for termination was influenced by the presumption that a child from sexual violence would be stigmatized:

When I realized I was pregnant I considered that pregnancy from sexual violence as a curse. I presumed that my children would stigmatize the child from sexual violence. Then I decided to terminate it.

An 18-year-old unmarried woman from the parenting group described the negative perceptions associated with children born from SVRPs and advocated for better treatment of both women and the children:

I delivered when I was too young and less strong. I was abandoned by my parents. Congolese women have experienced hardships. They are victims of war and need advocacy for they shouldn’t be considered like animals. Children born from rape should be treated like others born in regular situations; and all benefit from physical and mental care. Women should be respected and not abandoned.

A 40-year-old woman who was widowed and raising a child from sexual violence recounted the influence of both community rejection and peer rejection toward the child:

My big concern is that I have a child from sexual violence. The child was the cause of the rejection in the community. Also because I was raped twice. I tried to terminate that pregnancy without success. My first child was rejected in the community; she couldn’t take part and enjoy entertaining activities with other children.

### Theme 4: Trauma-informed desire for agency and advocacy for improved conditions for women

A prominent theme was the desire for agency toward social justice for women after sexual violence, for women with SVRPs, and for women in general. The following issues were highlighted by participants: call for an end to the violence in DRC, freedom to terminate an SVRP, an accessible and responsive justice system, and restoration of women’s dignity and value. As a 24-year-old married woman who had terminated an SVRP stated:

My worry is to stop sexual violence and Congolese women should be protected…Provide constantly women with medical care especially gynecological assistance and let women take the liberty of making termination in case they don’t need to have children delivered.

Women also offered suggestions about possible solutions to improve social and economic conditions for women with SVRPs and children born from SVRPs, including the need for medical care and housing. Among the suggestions, several women mentioned the importance of interventions among men to prevent rejection of women who had experienced sexual violence. A 28-year-old unmarried woman who had experienced two SVRPs in her lifetime (raising a child from the first SVRP and terminated the most recent SVRP) strongly advocated for women’s agency and education of men:

Women are abandoned, ill-treated. They are used as shields of enemies during wars…The first assistance is to stop wars, to restore dignity and respect of women in their communities, to provide advice and training to men whose wives were raped to prevent rejection.

Many participants described the suffering of women in DRC in relation to the ongoing conflict. Peacemaking was described as a first-step for improving the lives of women and children. A 40-year-old married respondent in the termination group noted:

My main concern about Congolese women is that they are abandoned, not considered and not respected. Also they suffer a lot over the war. Some are killed and others are victims of sexual violence. The first aid is to rebuild peace and to stop the war. Also to take care of children by providing them with education, medical care, and chiefly orphans and so on.

Finally, while many women in both the parenting and termination groups expressed despair for their present circumstances, many also strongly expressed a desire for women’s rights and agency:

Sexual violence, having a child, I know nothing about my future life. I can’t do any job apart from carrying burden, it’s a big problem. Congolese women should stand up and say no to sexual violence in order to be respected in Congo and overseas. As they are stigmatized, they need housing and care.

*21*-*year*-*old, not married, who terminated an SVRP*

I was so resentful of the rapist because he made me hopeless about marriage…My life is not very good. I have no family. My father and brothers were killed, my sister is a madwoman. I only do housework. I wonder if my child will go to school. I wonder if I will get married…Sexual violence should be stopped. The women’s dignity should be restored as a woman is the pillar of strength in a family.

*23*-*year*-*old, not married, raising a child from an SVRP*

## Discussion

The study provides qualitative data on psychosocial outcomes for women with SVRPs in eastern DRC. The data highlight a myriad of psychological sequelae and influences on psychological well-being, as women navigated complex, and often unstable, social environments after sexual violence and SVRPs. The interviews provided further details on the extent of loss of social supports, including abandonment and rejection by the spouses and families. Among participants, there was prominent mention of feeling excluded from society—an in/out group social identity. Prominent and poignant were the findings related to many respondents’ desire for agency and advocacy to advance the social conditions for women and survivors of violence in eastern DRC.

The qualitative data on emotional responses provide further context to the quantitative results from this study, which assessed symptom criteria for depression, post-traumatic stress disorder, anxiety, and suicidality, finding high prevalence of these mental health disorders among women with SVRPs [30, 31, 38]. Similar results were seen in other quantitative assessments of mental health outcomes among sexual violence survivors [3, 12]. The qualitative data provide additional information on concurrent social conditions and factors that are not readily captured by quantitative mental health assessments.

Although the qualitative study was not designed to look at differences between the parenting and termination groups, a number of observations arose. In regard to mental health, suicidal ideation or suicide attempts were described in the parenting group interviews, but not in the termination group interviews. Given the small sample sizes and the lack of design intended to look at differences in mental health outcomes between the two groups, it is difficult to interpret this finding. It is possible that termination of an SVRP provided relief and contributed to overall healing after a traumatic event, as noted in a study of rape-related pregnancies in the United States [39.] The quantitative analysis of mental health outcomes from this study did not directly compare the study groups; however, a similar proportion of women in each study group met screening criteria for depression and post-traumatic stress disorder, while a lower proportion in the termination group met screening criteria for anxiety or reported suicidality [30, 31].

A second observation of differences between the study groups in the qualitative study relates to description of marital status in the interviews. Many women in the termination group described being married in the interviews with the spouse’s reaction noted to play an important role in decision-making around the SVRP, whereas women in the parenting group commonly described being abandoned or separated in the interviews. It is important to note that married women were inadvertently under-sampled in the qualitative survey and it is impossible to know in all cases whether the marital separations resulted from sexual violence, the SVRP, a combination of the two, or from other unrelated factors. It is conceivable, however, that having terminated the SVRPs, some women may have been able to continue their marriages. These findings merit further exploration in future studies.

As in other studies of sexual violence survivors in DRC [7, 8, 13, 40], social stigmatization was commonly described in the interviews. This study’s previously published quantitative data showed that social stigmatization was a mediating factor of mental health outcomes and parenting relationships between women and their children born from SVRPs [30, 38], similar to the mediating role of stigma on mental health among sexual violence survivors described in other studies [14, 20]. Most of the interviews described enacted social stigma; however, internalized shame and social isolation were also depicted. There is a growing body of literature on the influences of stigma, negative social attitudes, and low levels of social integration and cohesion on trauma symptom severity and mental health following violence [19, 41–44]. Our study does not allow for conclusions about whether women who terminated SVRPs experienced more or less stigmatization within their communities since stigma from sexual violence, stigma from the SVRP, and stigma around pregnancy termination were not easily distinguishable in the interviews. Stigma related to pregnancy termination is described in other studies, but may also co-exist with a sense of reproductive agency among women [45]. Further research is needed on attitudes toward termination of SVRPs in this context.

The impact of stigma on mental health ultimately needs to be considered in the context of stressors and trauma related to conflict, poverty, and broader social conditions [14, 46]. Research shows that trauma is associated with poorer psychosocial functioning, independent of mental health disorders [47], and that multi-dimensional psychosocial care should be considered within the broader context of trauma-informed care [48]. Recent evidence also highlights the impacts of daily stressors on mental health, and suggests a sequenced approach to interventions, such that daily stressors are first addressed then followed by more specialized interventions to promote mental health [46]. According to Maslow’s hierarchy of needs [49], it is necessary to have basic needs met in order to advance psychological well-being. The interviews emphasized the importance of addressing the manifest needs related to shelter, food, education, and health care; suggesting that not only meeting those needs, but recognizing their significance, is crucial so that survivors are afforded an opportunity to address the trauma they have experienced.

The findings from this study have several important implications for programs and policies in DRC. Respondents identified the need for programs to prevent stigmatization of survivors of sexual violence and women with SVRPs and their children, including programs targeting men; similar to a previously reported program for sexual violence survivors in DRC [50]. Respondents also called for greater social support programs for women with SVRPs and their children, for restoring respect and dignity of women, and for social justice to end sexual violence and impunity. Other research notes that gender inequality results in inferior social status for women in terms of education, economics, justice, and personal autonomy; which is thought to contribute to the prevalence of sexual violence despite peace accords and sexual violence legislation in DRC [51, 52]. Future programs to promote accepting attitudes toward survivors of violence and women with SVRPs would need to address the cultural and social norms that support sexual violence as noted in other studies [53–56] and target inequitable gender norms [43].

Finally, while this study did not directly probe for post-traumatic growth, correlates are found in the interviews. Women reported a desire for agency and advocacy on behalf of themselves and other survivors of sexual violence and SVRPs; exemplifying dimensions of post-traumatic growth [18, 57]. The qualitative data also provide accounts of individual strength and resilience, which offers an opportunity for discourse on strategies for promoting psychosocial well-being for violence survivors. As an example, a psychosocial program among war-affected Darfuri women in Sudan integrated cultural conceptualizations, such as gender roles and religious expectations, and aimed to operationalize resilience and other psychosocial adaptation skills to meet the complexity of women’s experiences [58]. Further evidence suggests that there are cognitive, behavioral, and existential resilience factors that can be cultivated and should be included in interventions following exposure to trauma [59]. Psychosocial factors associated with resilience include optimism, cognitive flexibility, active coping skills, and maintaining a supportive social network [59]; many of these factors were evidenced in the interviews.

## Limitations

The study sample represents a systematic convenience sample of participants recruited using RDS; thus, the results may not be generalizable. While RDS is useful for sampling hard-to-reach populations, stigma and shame related to sexual violence may have prevented some individuals from participating in the study and women with alternative social experiences may not have been included in this sample. Given the cross-sectional study design, there are limitations in the ability to assess processes of recovery and psychological adaptation. The study was not designed to conduct follow-up interviews of participants, which would have allowed for further exploration of identified themes.

Furthermore, even though the interviews focused on the SVRP, it is not possible to know if the responses are related to the SVRP, to sexual violence, or to both. In some instances, women were asked about their experiences years after the event and the SVRP and there is the possibility of recall bias or limitations in the ability to articulate complex emotional responses years later. There are other potential sources of biases, including interviewer bias and a social desirability bias. Due to the sensitive nature of the subject, it is possible that the interviewers’ background and beliefs influenced their approach to the interviews, along with the participants’ willingness to open up to the interviewers. Additionally, biases may have been introduced through the processes of translation, coding and analysis; however, the translator and researchers had prior expertise and training. The interviews were not audio-recorded due to ethical concerns and the findings rely on notes taken at the time of the interview. While interviewers were trained on interviewing techniques, the lack of contextual data, such as non-verbal and verbal cues, may limit the depth of the data and data analysis.

## Conclusions

This qualitative study on psychosocial outcomes among women who experienced SVRPs in eastern DRC provides insight into how women navigated their social environments and lives following the SVRPs. The findings from the qualitative study, and in particular, the respondents’ suggested strategies, may be useful to inform future research, programs, and policies for women with SVRPs in eastern DRC. Future research should move beyond cross-sectional assessments to utilize innovative research methodologies to assess mechanisms of psychological adaptation among women with SVRPs. Multi-dimensional psychosocial programs for women with SVRPs should consider basic needs such as shelter, food, and health care within broader trauma-informed care. Participatory programming, guided by beneficiaries, could provide further avenues for agency to advance social conditions for women with SVRPs in eastern DRC.
